# Helvolic acid, an antibacterial nortriterpenoid from a fungal endophyte, *Xylaria* sp. of orchid *Anoectochilus setaceus* endemic to Sri Lanka

**DOI:** 10.1080/21501203.2014.892905

**Published:** 2014-03-25

**Authors:** Pamoda B. Ratnaweera, David E. Williams, E. Dilip de Silva, Ravi L.C. Wijesundera, Doralyn S. Dalisay, Raymond J. Andersen

**Affiliations:** a Department of Chemistry, University of Colombo, Colombo 03, Sri Lanka; b Department of Science and Technology, Uva Wellassa University, Badulla, Sri Lanka; c Department of Chemistry and Department of Earth, Ocean and Atmospheric Sciences, University of British Columbia, Vancouver, BC, Canada; d Department of Plant Sciences, University of Colombo, Colombo 03, Sri Lanka

**Keywords:** *Xylaria*, *Anoectochilus setaceus*, helvolic acid, antibacterial, endophytic fungi, rainforest

## Abstract

An endophytic fungus was isolated from surface sterilized leaf segments of *Anoectochilus setaceus,* an orchid endemic to Sri Lanka, and was identified as *Xylaria* sp. by morphological characters and DNA sequencing. Bioassay-guided chromatographic fractionation of the organic extract of a laboratory culture of this fungus led to the isolation of the known antibacterial helvolic acid. Helvolic acid was active against the Gram-positive bacteria, *Bacillus subtilis* [minimal inhibitory concentrations (MIC), 2 μg mL^−1^] and methicillin-resistant *Staphylococcus aureus* (MIC, 4 μg mL^−1^).

## Introduction

Endophytes are microorganisms that reside in the internal tissues of healthy plants, at least during one stage of their life cycle, without causing any apparent symptoms of disease or negative effects on their hosts (Suryanarayanan et al. 2009). Fungal endophytes are now recognized as a promising source of biologically active secondary metabolites often with novel structural features (Strobel & Daisy 2003; Aly et al. 2011). A comprehensive study has indicated that 51% of bioactive metabolites isolated from endophytic fungi were previously unknown compounds (Schulz et al. 2002). Although, increasingly, more attention is presently focused on the chemistry and the biological activities of endophytic fungal metabolites, a vast majority of the Earth's endophytic fungal biodiversity still remains completely unexplored. Thus, the investigation of endophytic fungi from unique ecological niches becomes important to realize the potential of this important resource.

*Anoectochilus setaceus* (Syn: *A. regalis*) is an endemic medicinal plant in Sri Lanka and has been traditionally used for treatment of snake bite poisoning (Jayaweera 1982). This plant belongs to family Orchidaceae and is currently listed as a vulnerable species in Sri Lanka facing a high risk of extinction in the wild (IUCN 2007). Up to now, there have been no reports on the biologically active metabolites of *A. setaceus* or the fungal endophytes associated with it. This article reports the isolation of an endophytic *Xylaria* sp. from *A. setaceus,* the investigation of antimicrobial properties of its organic extract and the bioassay-guided isolation and structure elucidation by nuclear magnetic resonance (NMR) and low-resolution mass spectra (LRMS) of helvolic acid, its antibacterial metabolite.

## Materials and methods

### Isolation of endophytes

Fresh healthy leaves of *A. setaceus* were collected from the Kanneliya forest reserve (6 09′–6 18′N & 80 19′–80 27′ E), Galle, Sri Lanka, in October 2011, brought to the laboratory inside a tightly sealed polythene bag and kept at room temperature under humid conditions. The leaves were used for isolation of endophytic fungi within 24 hours.

Prior to isolation of endophytes, the plant material was surface sterilized with 70% ethanol and 5% sodium hypochlorite according to the published procedure described by Radji et al. (2011). Squares of about 0.5^2^ cm obtained from the surface sterilized leaves were placed on potato dextrose agar (PDA) (Himedia) medium in Petri dishes. The dishes were thereafter sealed and incubated at room temperature (30°C) until the growth of endophytic fungi was observed. After 7 days, small plugs having the mycelium growing out of the leaf edges were cut and transferred onto fresh PDA dishes. Serial subculturing was done until pure cultures were obtained. The isolated pure fungus was stored as PDA slants in glycerol.

### Identification of the endophytic fungus

The isolated endophytic fungus was initially identified through microscopic examination of colony morphological and reproductive characteristics using slide cultures. Fungal DNA was extracted in the laboratory using the protocol of Kariyawasam et al. (2012). The extracted DNA was subjected to the polymerase chain reaction (PCR) using primers ITS1 and LR3R. Amplified DNA was subjected to DNA sequencing and this DNA sequence was compared with already existing DNA sequences in NCBI GenBank (http://www.ncbi.nlm.nih.gov.blast). PCR and DNA sequencing was done by the GeneTech Institute, Sri Lanka. The acquired gene sequence was submitted to the NCBI GenBank database and an accession number was obtained.

### Extraction and screening for antimicrobial activities

The isolated endophytic fungus was grown on 150 PDA medium Petri dishes (100 × 20 mm) for 28 days at room temperature. Each dish had 15 mL of PDA. At the end of the incubation period, the medium together with the fungal mycelium in all dishes was cut into small pieces and immersed in ethyl acetate (700 mL) for 48 hours and filtered through glass wool. This extraction process was repeated thrice. The filtrates were combined and the organic solvent was evaporated to dryness under reduced pressure at room temperature using a rotary evaporator (BUCHI-R-200). A sample of freshly collected aerial parts of *A. setaceus* was also extracted with methanol/dichloromethane, 1:1 mixture and concentrated as described earlier for the fungus extract. The weights of the two crude samples were obtained using an analytical balance.

The crude extracts of the fungus and the plant were tested for antimicrobial activities against two Gram-positive bacteria, *Bacillus subtilis* (UBC 344), methicillin-resistant *Staphylococcus aureus* (MRSA, ATCC 33591), two pathogenic Gram-negative bacteria, *Escherichia coli* (UBC 8161) and *Pseudomonas aeruginosa* (ATCC 27853) and a pathogenic fungus *Candida albicans* (ATCC 90028) at 50 μg per disc using the agar disc diffusion method (National Committee for Clinical Laboratory Standards 2003).

### Fractionation, isolation and structure elucidation of the bioactive component

To isolate the principal bioactive component(s) from the complex mixture of the fungal extract, a series of bioassay-guided purification steps were performed. The crude extract (400 mg) was first subjected to Sephadex LH-20 size exclusion chromatography (2.5 × 175-cm column) with methanol as the eluent. Resulting fractions were combined according to the thin layer chromatography (TLC) profiles and the combined fractions were tested for antibacterial activity. The most active fraction was next chromatographed once again on Sephadex LH-20 (2.5 × 175-cm column) with ethyl acetate/methanol/water in 20:5:2 ratios as the eluting solvent. The resulting active fraction (15 mg) was finally purified on normal phase silica (2 × 30-cm column) using gradient elution (1% to 20% methanol:dichloromethane) to obtain the active component. The structure elucidation of the isolated compound was done using NMR and mass spectral data. ^1^H, ^13^C and 2D NMR spectral data sets were obtained using a Bruker AVANCE 600-MHz spectrophotometer with cryoprobe, while mass spectroscopy (MS) data of the compound was obtained using Bruker Esquire-LC electrospray spectrophotometer.

### Antibacterial activity of the isolated pure compound

The pure compound was tested for antimicrobial activities against two Gram-positive bacteria, *B. subtilis* (UBC 344) and MRSA (ATCC 33591). The minimum inhibitory concentrations (MICs) were determined using broth micro-dilution method according to National Committee for Clinical Laboratory Standards (2002) with modification using Mueller Hinton Broth as the medium. The commercial antibacterial agents polymyxin B and rifamycin were used as positive controls.

## Results and discussion

### Isolation and identification of the endophytic fungal strain

**Figure 1.**
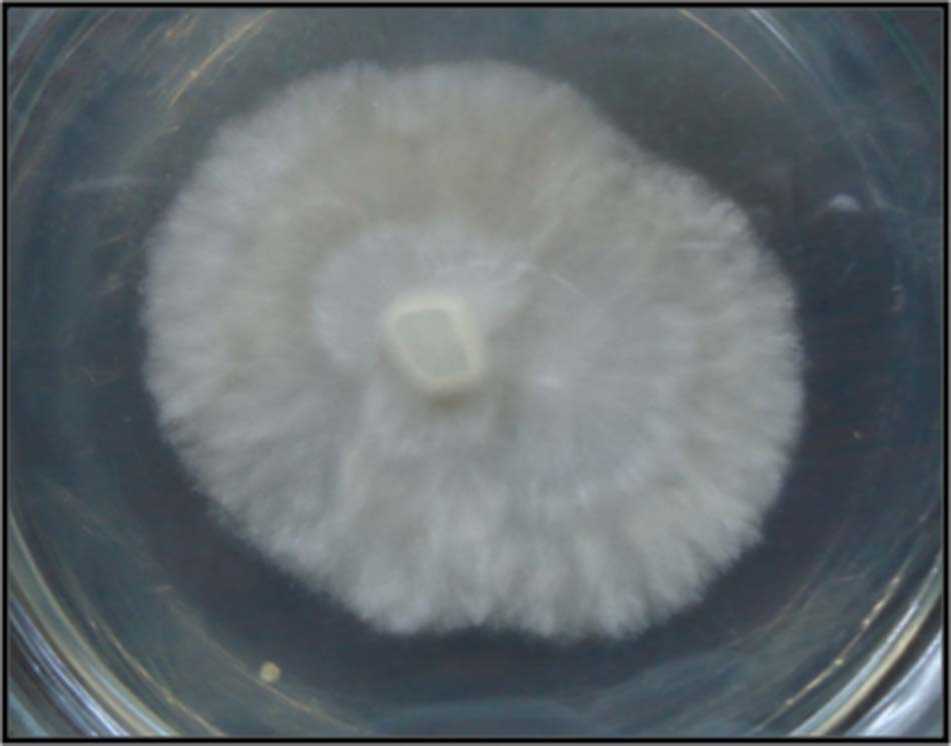
Endophytic WR1 fungus.

**Figure 2.**
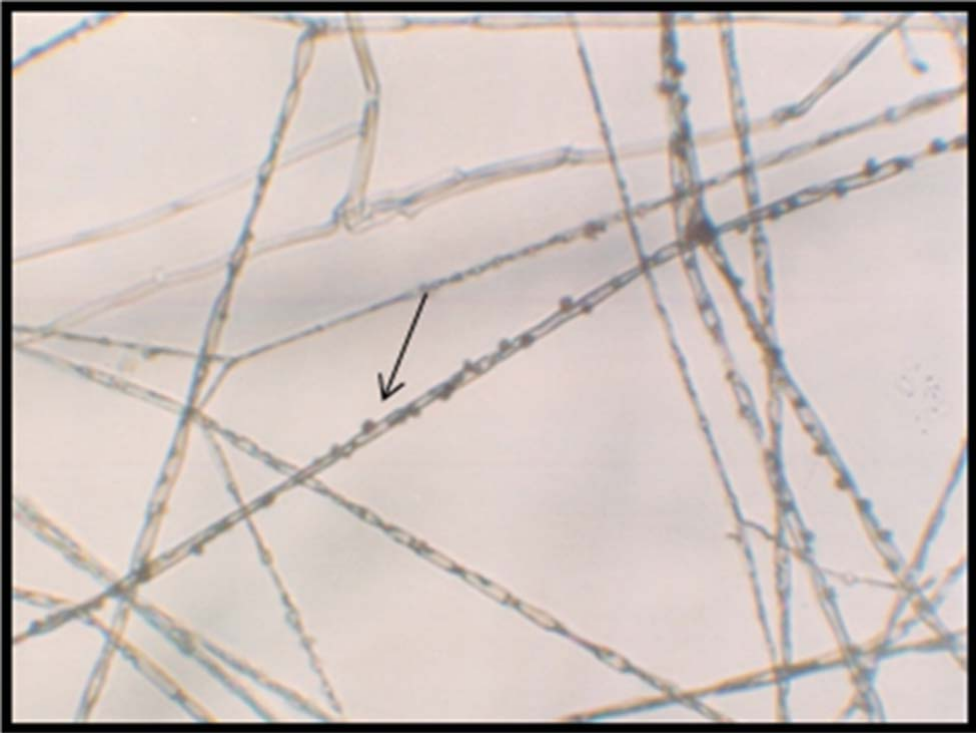
Septate hyphae with pigmented crystals (×400).

On PDA medium, the isolated fungal culture has an off-white colour with threadlike mycelia with wavy margins (Figure 1). After approximately 25 days, it started to secrete a light yellowish pigment. The slide cultures prepared from this fungus showed septate hyphae with pigmented crystals along them (Figure 2). Molecular identification techniques were used to determine the identity of the fungus to generic level. A GenBank search revealed that several species of *Xylaria* as the closest matches with sequence identities ranging from 99% to 97%. The most similar sequence was that of *Xylaria* sp. SGLAf34 (accession number EU715609), an endophytic fungus isolated from Mexican yew at the Sierra Gorda Biosphere Reserve (Soca-Chafre et al. 2009), followed by several other sequences of *Xylaria* sp. (Table 1). On the basis of its 18S ribosomal RNA gene, partial sequence; internal transcribed spacer 1, 5.8S ribosomal RNA gene, and internal transcribed spacer 2, complete sequence and 28S ribosomal RNA gene, partial sequence, it can be concluded that the fungus WR1 belongs to the genus *Xylaria.* The NCBI GenBank accession number for the gene sequence of WR1 fungus is JX523620 (GI:408455748).

**Table 1.** Comparison of the ITS and 18SrDNA sequence obtained from WR1 (JX523620) to their closest relatives available in the NCBI nucleotide sequences database.

Accession number	Closest relatives	Identity (%)	Sequences
EU715609	*Xylaria* sp. SGLAf34	99	18SrDNA
JN418791	*Xylaria* sp. E10212e	99	ITS
FJ612908	Fungal sp. AR1Z B08	99	18SrDNA
AY315407	Xylariaceae sp. F9	99	ITS
HM992496	Xylariaceae sp. E76061	98	18SrDNA
EU715606	*Xylaria* sp. SGLAf26	97	18SrDNA

Endophytic Xylariaceae have a worldwide distribution from tropical forests to arctic environments and have been reported from various plants, including conifers, monocots, dicots, ferns and lycopsids (Brunner and Petrini 1992). However, this is the first report of an endophytic *Xylaria* sp. from the endemic orchid, *A. setaceus.* The genus *Xylaria* shows great variation in morphology and only a small number of species produce species-specific morphological features in culture, which makes it virtually indistinguishable, thus making the identification of endophytic *Xylaria* to the species level very difficult. Therefore, past studies have relied on molecular techniques to find out the relationships of species within the genus (Brunner & Petrini 1992; Liu et al. 2008). In the current investigation, the pigmented crystals along the hyphae that were observed in slide cultures made this fungus very unusual and also difficult to identify to the species level. However, as this fungus was isolated from an endemic orchid, found in a unique rainforest setting in Sri Lanka, it may imply that this fungus belongs to a new species of *Xylaria.* More precise taxonomic identification of this fungus may require more prudent molecular techniques, expansion of fungal genomic database and further studies using several cultures of the same fungus.

### Extraction and screening for antimicrobial activities

The ethyl acetate extraction of the cultured fungus and the medium yielded 400 mg of crude fungal extract while the extraction of the aerial parts of *A. setaceus* yielded 150 mg. The agar disc diffusion assay results revealed that the crude ethyl acetate extract of the endophytic *Xylaira* sp. was active against Gram-positive bacteria with inhibition zones of 14 and 13 mm diameter at 50 μg/disc for *B. subtilis* (UBC 344) and MRSA (ATCC 33591), respectively. The crude fungal extract was inactive against the two tested pathogenic Gram-negative bacteria, *E. coli* (UBC 8161) and *P. aeruginosa* (ATCC 27853), and the pathogenic fungus *C. albicans* (ATCC 90028). The extract of *A. setaceus* was inactive against all of the tested bacteria at 50 μg per disc.

### Isolation and structure elucidation of the active compound

**Figure 3.**
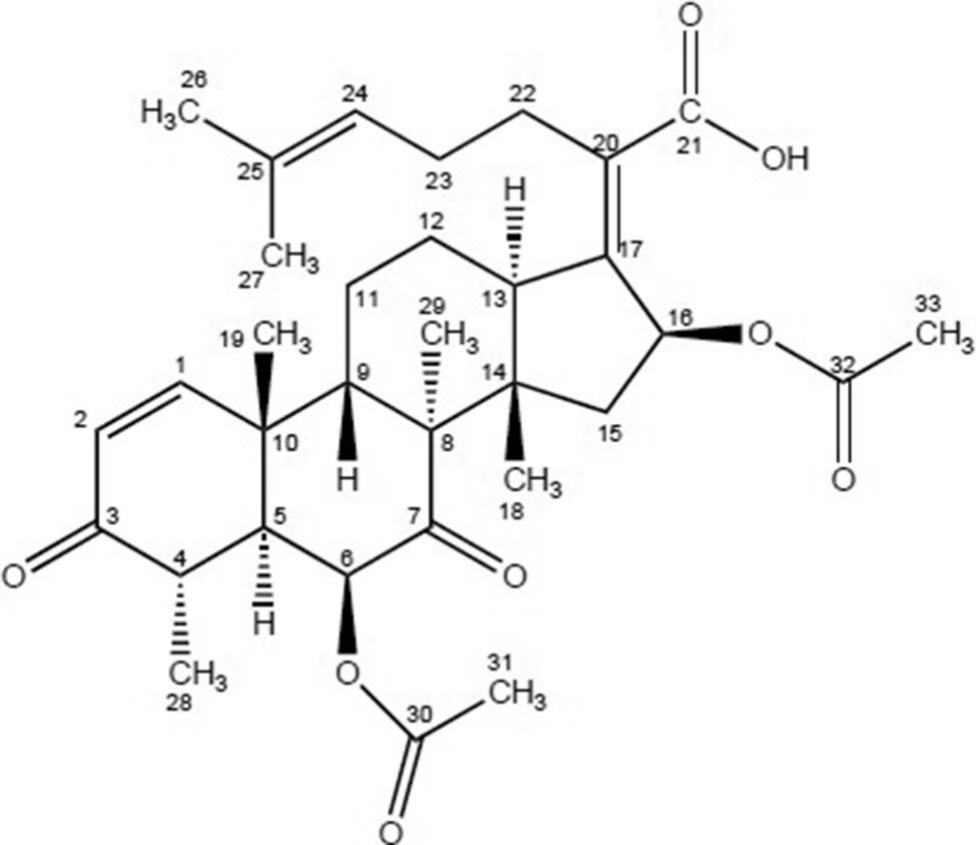
Chemical structure of helvolic acid.

Size exclusion chromatography of the crude extract (400 mg) followed by silica gel chromatography led to the isolation of the active compound (white needle-like crystals; 3 mg), which gave a mass of *m/z* 567.4 for the (*M* – 1) ion in its low-resolution electrospray ionization mass spectrum, corresponding to a molecular formula of C_33_H_44_O_8_. Analysis of ^1^H and ^13^C NMR as well as 2D NMR (COSY, HSQC, HMBC, ROESY) spectral data in dimethyl sulfoxide (DMSO) revealed that the structure of the active compound (Figure 3) matches that of the known nortriterpenoid helvolic acid (Fujimoto et al. 1996). Both ^1^H and ^13^C NMR data obtained for helvolic acid were consistent with those reported by Fujimoto et al. (1996). A comparison of ^13^C NMR values obtained in the present study for helvolic acid with already reported data (Fujimoto et al. 1996) is given in Table 2. The ^1^H NMR values obtained in the present study are 7.39 (d, 10.2), 5.75 (d, 9.96), QC, 2.74 (dq 12.36, 6.75), 2.63 (d, 11.28), 5.04 (s), QC, QC, 2.45 (dd, 12.6, 2.2), QC, 1.66 (m), 1.2 (m), 1.72 (dd, 13.11, 4.5), 2.39 (d, 12.36), QC, 1.91 (d, 14.7), 2.41 (dd, 13.68, 2.64), 5.63 (d, 8.4), QC, 0.79 (3H, s), 1.38 (3H, s), QC, QC, 2.42 (2H, m), 2.3 (2H), 5.1 (t, 7.14), QC, 1.56 (3H, s), 1.64 (3H, s) 1.11(d, 5.58), 1.09 (3H,s), QC, 1.87 (3H, s), 2.07 (3H, s).

**Table 2.** Comparison of ^13^C NMR data of helvolic acid from the present study in DMSO with published data in CDCl_3_ (Fujimoto et al. 1996).

	^13^C δ (ppm)			^13^C δ (ppm)			^13^C δ (ppm)	
	Present study	Published values	C#	Present study	Published values	C#	Present study	Published values
1	158.42	157.3	12	25.50	25.9	23	28.00	28.3
2	126.86	127.8	13	46.08	49.4	24	123.24	122.8
3	200.88	201.4	14	45.51	46.6	25	131.67	132.9
4	40.04	40.4	15	40.34	40.6	26	17.64	17.8
5	48.01	47.2	16	73.04	73.5	27	25.33	25.7
6	73.08	73.8	17	144.34	147.6	28	12.29	13.1
7	209.26	208.8	18	17.64	17.9	29	17.69	18.3
	52.25	52.7	19	27.17	27.5	30/32	169.10/169.68	168.9/170.3
9	41.19	41.7	20	131.02	130.5	31/33	20.38/20.44	20.7/20.5
10	37.70	38.2	21	171.01	174.2			
11	23.1	23.9	22	28.26	28.6			

### Antimicrobial activities of helvolic acid from *Xylaria* sp. WR1

Helvolic acid exhibited strong antibacterial activities against the Gram-positive *B. subtilis* (MIC: 2 μg mL^−1^) and MRSA (MIC: 4 μg mL^−1^). MIC of the positive controls, polymyxin B is 8 μg mL^−1^ for *B. subtilis* while 0.015 μg mL^−1^ for rifamycin against MRSA.

Previous studies have reported that helvolic acid exhibits antibacterial activities mainly against Gram-positive bacteria (Chain et al. 1943; Qin et al. 2009). The MICs against the Gram-positive bacteria reported in these studies are in the range 4–16 mg L^−1^ and are higher than in the current study. In another study (Zhang et al. 2008), helvolic acid has shown activity against *E. coli* at a concentration of 14.49 μM, which shows a contradictory result.

Fungi of the genus *Xylaria* are known to be very diverse with respect to their chemical constituents and biological activities. Previous studies have reported polyketides, cytochalasins, terpenoids, coumarins, xyloketals, cyclopeptides and xanthones from various *Xylaria* sp., thus demonstrating antimicrobial, antitumour and acetylcholinesterase (AChE) inhibitory activities (Espada et al. 1997; Lin et al. 2001; Joong-Hyeop et al. 2005; Li et al. 2010; Oliveira et al. 2011). Yet, this is the first study to report the nortriterpenoid antibiotic helvolic acid from an endophytic *Xylaria* sp. According to previous studies, helvolic acid has been isolated from *Aspergillus fumigatus, Acrocylindrium oryzae, Cephalosporium caerulens, Metarhizium anisopliae, Pichia guiliermondii* and a few other fungal species (Chain et al. 1943; Okuda et al. 1966; von Daehne et al. 1968; Wei 1979; Fugimoto et al. 1996; Tschen et al. 1997; Lee et al. 2008; Zhang et al. 2008; Feng & Ma 2010; Zhao et al. 2012).

According to Strobel et al. (2004), tropical rainforests are most diverse and crowded ecosystems and requires individual organisms to develop novel biosynthetic pathways that are beneficial for their survival. In the backdrop that the organic extracts of *A. setaceus* was inactive against the tested bacteria, the mutualistic association of an endophytic fungus capable of producing the strong antibacterial helvolic acid would be expected to confer an advantage to *A. setaceus* to wade off probable microbial attacks in the natural setting.
